# Contact force-guided catheter ablation for the treatment of atrial fibrillation: a meta-analysis of randomized, controlled trials

**DOI:** 10.1590/1414-431X20155127

**Published:** 2016-02-02

**Authors:** Z. Qi, X. Luo, B. Wu, H. Shi, B. Jin, Z. Wen

**Affiliations:** Department of Cardiology, Huashan Hospital, Fudan University, Shanghai, China

**Keywords:** Atrial fibrillation, Contact force sensing, Meta-analysis, Randomized, controlled trials

## Abstract

Contact force (CF) sensing technology allows real-time monitoring during catheter ablation for atrial fibrillation (AF). However, the effect of CF sensing technology on procedural parameters and clinical outcomes still needs clarification. Because of the inconsistent results thus far in this area, we performed a meta-analysis to determine whether CF sensing technology can improve procedural parameters and clinical outcomes for the treatment of AF. Studies examining the benefits of CF sensing technology were identified in English-language articles by searching the MEDLINE, Web of Science, and Cochrane Library databases (inception to May 2015). Ten randomized, controlled trials involving 1834 patients (1263 males, 571 females) were included in the meta-analysis (681 in the CF group, 1153 in the control group). Overall, the ablation time was significantly decreased by 7.34 min (95%CI=-12.21 to -2.46; P=0.003, Z test) in the CF group compared with the control group. CF sensing technology was associated with significantly improved freedom from AF after 12 months (OR=1.55, 95%CI=1.20 to 1.99; P=0.0007) and complications were significantly lower in the CF group than in the control group (OR=0.50, 95%CI=0.29 to 0.87; P=0.01). However, fluoroscopy time analysis showed no significantly decreased trend associated with CF-guided catheter ablation (weighted mean difference: -2.59; 95%CI=-9.06 to 3.88; P=0.43). The present meta-analysis shows improvement in ablation time and freedom from AF after 12 months in AF patients treated with CF-guided catheter ablation. However, CF-guided catheter ablation does not decrease fluoroscopy time.

## Introduction

Radiofrequency catheter ablation (RFCA) is a potentially curative method for treatment of atrial fibrillation (AF). The 2012 guidelines recommend catheter ablation in cases of symptomatic paroxysmal AF or for those intolerant to antiarrhythmic medication (1). Pulmonary vein isolation remains the cornerstone of the strategy for all catheter ablation procedures (2). Probably the most important limitation of RFCA for AF is the rate of recurrence owing to electrical reconnection of the pulmonary veins (3,4). This recurrence is associated with poor quality of life; and, if these reconnections were eliminated, long-term success rates would improve (5,6).

Contact force (CF), which occurs between the catheter tip and target tissue, is a crucial determinant of the lesion characteristics of radiofrequency. Several studies (7,8) have reported that the use of a CF-guided catheter may significantly improve procedural parameters and clinical outcomes. However, CF sensing technology does not improve clinical outcomes in patients with paroxysmal AF (9). Therefore, we performed a meta-analysis to determine whether the use of CF sensing technology can improve procedural parameters and clinical outcomes in the treatment of AF.

## Material and Methods

### Study search strategy

Studies that investigated the benefits of CF sensing technology were identified in English-language articles by searching the MEDLINE, Web of Science, and Cochrane Library databases (inception to May 2015). We used the search terms: "atrial fibrillation", "contact force", "pulmonary vein isolation", and "catheter ablation".

### Inclusion criteria

We identified eligible articles on the basis of the following inclusion criteria: 1) study design (randomized, controlled trials), 2) target population (AF patients who underwent RFCA), 3) intervention (studies involving CF-guided catheter ablation), and 4) outcomes (procedural parameters and clinical outcomes were examined in the target population). When multiple studies shared the same subject population, we included only the most recent study.

### Data extraction

Two reviewers independently extracted data from all eligible studies fulfilling the inclusion criteria. Disagreement was resolved by discussion between the two reviewers. We extracted the following data from the included studies: the first author, publication date, region of origin, number of patients, ablation device, study design, and baseline characteristics. For data that were not provided in the main text, the required information was obtained in part from the supplementary online appendix.

### Statistical methods

The Cochrane Collaboration meta-analysis review methodology was used for this study. Continuous variables with a normal distribution are reported as means±SD. The effects of continuous variables were evaluated as weighted mean differences (WMDs). Odds ratios (ORs) were used as summary statistics for discontinuous variables. The presence of heterogeneity across studies was evaluated. P≤0.10 was considered to be significant for statistical heterogeneity (10). All statistical analyses were performed with RevMan version 4.2.2, which is available from the Cochrane Collaboration website (http://www.cochrane.org/cochrane/hbook/htm).

## Results

### Identification of studies

A total of 364 potentially eligible citations were identified using our search strategy. After the initial screening, 33 relevant articles were selected for further review. Among these, 23 articles were excluded according to the inclusion criteria. Ten randomized, controlled trials involving 1834 patients were eventually identified and included in the meta-analysis (681 in the CF group, 1153 in the control group) (11 12 13 14 15 16 17 18 19 20).

### Characteristics of the studies

Table 1 shows the characteristics of the 10 clinical trials that were published between 2012 and 2015. The duration of follow-up ranged from the time of discharge to 12 months. Baseline characteristics of the two study groups were well balanced with respect to baseline features. In the CF group, the SmartTouch (Biosense Webster, USA) or Tacticath (Endosense SA, Switzerland) ablation catheter was used, while a standard catheter or cryoballoon catheter was used in the control group.

**Table:**
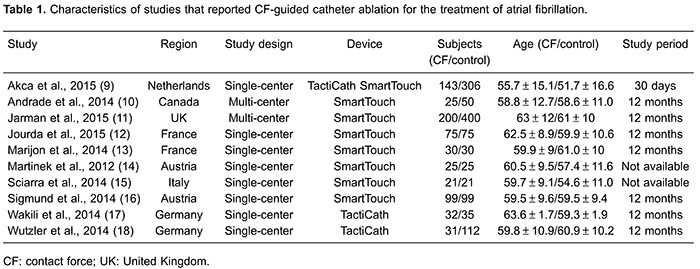

### Procedural parameters

**Figure 1:**
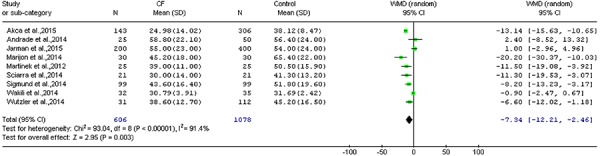
Ablation time in the CF group compared to the control group with catheter ablation of atrial fibrillation (WMD=-7.34; 95%CI=-12.21 to -2.46; P=0.003; Z test). CF: contact force; WMD: weighted mean difference. For reference details, see Table 1.

**Figure 2:**
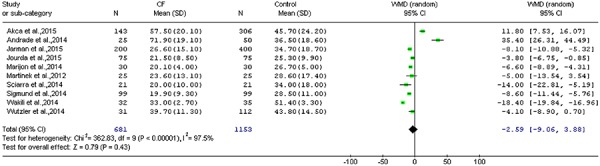
Forest plot of fluoroscopy time meta-analysis of contact force (CF) sensing technology in catheter ablation of atrial fibrillation (weighted mean difference [WMD]: -2.59; 95%CI=-9.06 to 3.88; P=0.43; Z test). For reference details, see Table 1.

Table 2 shows the main results of the pooled WMDs and ORs in the meta-analysis. The procedural parameters mainly included ablation time, overall duration of the procedure, and fluoroscopy time. Data on ablation time were available in nine randomized, controlled trials. CF sensing technology was associated with a significantly decreased ablation time (WMD=-7.34; 95% confidence interval [CI]=-12.21 to -2.46; P=0.003; Figure 1). An analysis of overall duration of the procedure indicated that CF-guided catheter ablation advantageously reduced the overall duration (WMD=-19.43; 95%CI=-29.61 to -9.26; P=0.0002; Table 2). However, the WMD estimate indicated that CF sensing technology did not significantly decrease the fluoroscopy time compared with in the control group (WMD=-2.59; 95%CI=-9.06 to 3.88; P=0.43; Figure 2).

**Table:**
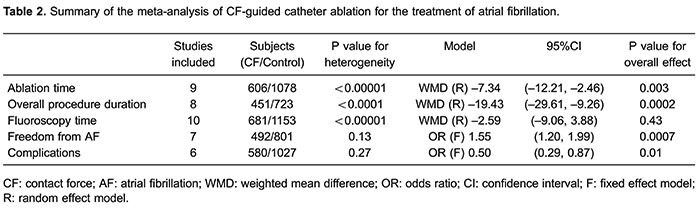

### Clinical outcomes

**Figure 3:**
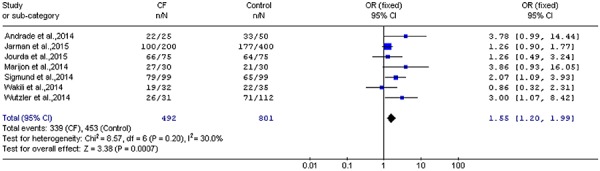
Cumulative OR estimate of freedom from atrial fibrillation after 12 months in the contact force (CF) group compared to the control group (OR=1.55; 95%CI=1.20 to 1.99; P=0.0007; Z test). For reference details, see Table 1.

**Figure 4:**
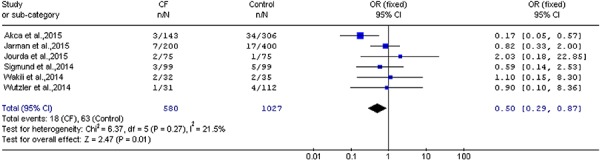
Comparison of complications in the contact force (CF) group compared to the control group (OR=0.50; 95%CI=0.29 to 0.87; P=0.01; Z test). For reference details, see Table 1.

Efficacy analysis of freedom from AF after 12 months showed that CF-guided catheter ablation was associated with a significant improvement in freedom from AF (OR=1.55; 95%CI=1.20 to 1.99; P=0.0007; Figure 3). Additionally, CF sensing technology significantly decreased complications (OR=0.50; 95%CI=0.29 to 0.87; P=0.01; Figure 4).

### Sensitivity analysis

A single study involved in the meta-analysis was deleted each time to reflect the influence of the individual data set on the pooled WMDs or ORs. We found that the corresponding WMDs or ORs were not materially altered, which indicated that our results were statistically robust.

### Publication bias

Begg's funnel plot and Egger's test were performed to determine the publication bias of the literature. Funnel plot analysis did not show any evidence of obvious asymmetry and possible publication bias should not have substantially influenced the results of this meta-analysis.

## Discussion

CF-guided catheter ablation is a new technology for ablating AF. Therefore, the effect of this technology on procedural parameters and clinical outcomes needs to be determined. To circumvent the reversibility of pulmonary vein isolation, ensuring that first-time RF lesions are optimal and result in permanent myocardial tissue damage is important (21). Optimal contact between the catheter tip and tissue during RF ablation appears to be important for providing the best radiofrequency ablation lesions (22 23 24). Currently, several parameters are used to gauge adequate contact, such as pure tactile feedback, impedance, and intracardiac electrograms. Nevertheless, these parameters are unable to precisely ensure CF because of differences in the catheter shaft, deflection, torque, and types of sheaths that are used in the procedure. Additionally, electrogram reduction is a particularly poor predictor of transmurality (25 26 27).

Appropriate CF is important for catheter ablation because insufficient CF results in ineffective delivery of radiofrequency, whereas excessive CF can cause collateral tissue injury. Continuous CF monitoring has many advantages for catheter ablation. First, this monitoring ensures that the catheter is correctly placed, including application and direction. Second, continuous CF monitoring can monitor the catheter's movements in real time. Therefore, taking the abovementioned factors into account, using CF sensing technology during RFCA for AF is a useful development.

Our meta-analysis demonstrated that CF sensing technology could not only reduce ablation time and the overall duration of procedure, but also improve freedom from AF after 12 months. Although CF-sensing did not reduce fluoroscopy time, we believe that this will eventually be achieved as operators gain more experience in the future. With regard to operators, real-time CF measurements will allow them to favorably modulate the CF and thus the lesion size. Therefore, a point with a CF that is too low or too high because of difficulty in catheter positioning could potentially be compensated for by changing the radiofrequency power or the duration (28).

Even though we performed a pooled analysis, which included 10 randomized, controlled trials, some caution is needed in the interpretation of our results. Overall, the meta-analysis was not performed using individual patient data. Therefore, prespecified data were partly extracted from the studies for analysis. Several studies with a small sample size were included, which were likely to involve selected cohorts of patients and operators. Additionally, potential heterogeneity among the clinical trials due to varying inclusion criteria, the definition of variables, and different ablation devices, cannot be excluded.

In summary, the present meta-analysis showed improvement in ablation time and freedom from AF after 12 months in AF patients treated with CF-guided catheter ablation. However, CF-guided catheter ablation does not decrease fluoroscopy time. Because of the relatively small sample size of our meta-analysis, larger-scale, prospectively designed, randomized, double-blinded trials should be carried out to clarify the potential benefits of CF-guided catheter ablation for the treatment of AF.
